# Analysis of factors influencing axial length changes in adolescents wearing corneal reshaping lenses

**DOI:** 10.5935/0004-2749.2025-0074

**Published:** 2025-06-24

**Authors:** Guangbin Zhong, Ruinian Zheng, Linyi Luo

**Affiliations:** 1 Department of Ophthalmology, The Tenth Affiliated Hospital of Southern Medical University (Dongguan People’s Hospital), Dongguan, China; 2 Dongguan Institute of Clinical Cancer Research, Dongguan Key Laboratory of Precision Diagnosis and Treatment for Tumors, The Tenth Affiliated Hospital of Southern Medical University (Dongguan People’s Hospital), Dongguan, China; 3 Department of Science and Education, The Tenth Affiliated Hospital of Southern Medical University (Dongguan People’s Hospital), Dongguan, China

**Keywords:** Orthokeratology, Contact lens, Myopia, Adolescent, Axial length, eye

## Abstract

**Purpose:**

This study aimed to identify factors influencing axial length changes in adolescents wearing orthokeratology lenses.

**Methods:**

A retrospective analysis was conducted on 84 adolescents (aged 9-17 yr) who wore orthokeratology lenses at our hospital. Axial length changes were calculated as the difference between the first and last visits. Patients were categorized into two groups based on axial length change: lower-than-average and higher-than-average. Data on sex, age at orthokeratology lens initiation, family history, initial equivalent spherical lens value, initial cylindrical lens value, initial average K value, and initial axial length were collected. Univariate and mixed-effects model analyses were performed to assess their influence on axial length changes.

**Results:**

Age (p<0.05) and initial equivalent spherical value (p<0.05) were significant predictors of axial length changes in both eyes and the left eye. For the right eye, only age was a significant factor (p<0.05). The mixed-effects model revealed that the difference between the left and right eyes, duration of orthokeratology lens use, age, initial equivalent spherical lens value, and initial axial length significantly influenced axial length changes in adolescents (p<0.05).

**Conclusion:**

The factors influencing axial length changes in adolescents wearing orthokeratology lenses differ between the left and right eyes. These changes depend on the duration of lens wear, age, initial equivalent spherical lens value, and initial axial length. This study provides a theoretical basis for evaluating the clinical efficacy of orthokeratology lenses in managing myopia progression in adolescents.

## INTRODUCTION

Myopia, a type of ametropia, primarily results from excessive refractive power of the cornea or lens or an elongated axial length, with the latter accounting for over 95% of cases^(1)^. Pathological myopia can lead to severe complications such as myopic macular degeneration, open-angle glaucoma, and even blindness in extreme cases. Research suggests that myopia arises from a complex interplay between environmental factors and genetic predisposition. Globally, the increasing use of electronic devices among young individuals and the rising academic demands have contributed to the growing prevalence of myopia. Consequently, identifying safe and effective interventions for myopic adolescents is of significant clinical importance.

Orthokeratology (Ortho-K) lenses-also known as OK lenses, visual shaping therapy, or corneal refractive therapy (CRT)-offer a non-surgical approach to temporarily correct myopia by reshaping the cornea. These rigid, gas-permeable lenses flatten the central cornea, altering its curvature and mitigating myopic refraction, thereby improving visual acuity^(2^,^3)^. Ortho-K lenses function by flattening the central cornea, increasing the curvature diameter, and reducing the corneal curvature force through a reverse geometric design and highly oxygen-permeable materials. This process mitigates myopic refraction and restores visual acuity. In addition to controlling myopia progression in adolescents, Ortho-K lenses eliminate the inconvenience of wearing eyeglasses. However, traditional Ortho-K lenses have notable drawbacks, including suboptimal parameter design, insufficient material permeability, and poor predictability.

Research indicates that increased axial length is a primary factor in myopia progression. A previous study involving 60 adult eyes with high myopia (axial length ≥26 mm) demonstrated axial elongation of 0.03-0.06 mm per year over 6 yr. Additionally, visual impairment correlates more strongly with axial length than with refractive error. Consequently, axial elongation is now a key parameter in clinical trials aimed at slowing myopia progression.

This study compared data from two groups: one with lower-than-average axial length changes and another with higher-than-average changes. The objective was to assess the impact of Ortho-K lens usage on axial length variations in adolescents, providing a theoretical reference for evaluating the clinical efficacy of these lenses in myopic adolescents.

## METHODS

### Subjects

This retrospective study analyzed data from 84 adolescents who wore Ortho-K lenses in the Tenth Affiliated Hospital of Southern Medical University (Dongguan People’s Hospital) between January 2017 and December 2019. The participants, aged 9-17 yr (mean age: 12.41 ± 2.18 yr), included 49 males and 35 females. Inclusion criteria included age ≥9 yr, intraocular pressure <21 mmHg, myopia diopter <-6.00 DS, with-the-rule astigmatism <1.75 DC, against-the-rule astigmatism <0.75 DC, ability to wear and remove Ortho-K lenses properly, and adherence to daily lens care requirements. Exclusion criteria encompassed history of ophthalmic diseases (e.g., severe myopia, keratoconus, chronic and dacryocystitis), active ocular surface conditions, corneal abnormalities, or fundus lesions, ocular hypertension, systemic diseases affecting vision (e.g., immune disorders and diabetes), use of atropine or other vision-altering medications. All patients and their families provided informed consent for Ortho-K treatment and study participation. The study was approved by the Ethics Committee of The Tenth Affiliated Hospital of Southern Medical University (Dongguan People’s Hospital).

### Wearing Ortho-K lenses

All patients underwent a comprehensive ophthalmic examination, which included assessments of visual acuity, external eye structure, anterior segment, corneal topography, intraocular pressure, and mydriatic fundus. Additionally, mydriatic and comprehensive optometry tests were conducted. These tests included the initial maximum plus to maximum visual acuity (MPMVA) of each eye, the initial red-green balance, axial and degree of precise astigmatism using the Jackson cross cylinder (JCC), a second MPMVA assessment, binocular MPMVA, and binocular balance detection. Measurements of basic axial length and corneal endothelial cell density were also recorded. Mydriatic subjective refraction, corneal K value, corneal aspheric coefficient, and iris diameter were determined. Based on these parameters, a customized trial lens was prepared.

Following the standard protocol for Ortho-K lens fitting, patients who met the eligibility criteria were enrolled, and relevant medical records were established. A lens trial was conducted, and those who demonstrated suitability were fitted with customized lenses by professional optometrists. The lenses were fabricated and adjusted according to test results. Upon delivery, patients were instructed on lens care, application, and removal, along with necessary precautions. Regular outpatient follow-ups were scheduled. The Ortho-K lenses used in this study were Mengdaiwei Ortho-K lenses (Hefei OVCTEK Company), made of Boston XOP material with an oxygen permeability coefficient of 100 × 10⁻^^11^^ (cm^2^.mLO₂)/(s.mL.mmHg). These lenses featured a conventional four-zone, five-arc design with diameters ranging from 10.2 to 11.0 mm and corneal curvature values of 42.00-45.5D. The participants were evaluated following the standard fitting procedure for Ortho-K lenses. The final prescription was determined based on center positioning, mobility, fluorescence ring, and edge arc. All patients wore Ortho-K lenses for 8-10 h each night. Follow-up examinations were conducted on the first day, 1 week, 1 month, 3 months, and 6 months after initiation and subsequently every 6 months. The assessments included uncorrected visual acuity, intraocular pressure, small pupil optometry, and corneal curvature. Corneal health was evaluated using a slit lamp microscope, and axial length was measured at 6-month intervals.

### Observation indicators

The following baseline characteristics were recorded: sex, age at initial Ortho-K lens use, family history of myopia, initial equivalent spherical lens value, initial cylindrical lens value, initial average K-value, and initial axial length. Axial length change was calculated at the study’s conclusion. Patients were categorized into two groups based on whether their axial length change was above or below the study’s average. Axial length was measured using the IOL-Master 500.

### Statistical analysis

Data were expressed as mean ± standard deviation. Statistical analyses were performed using SPSS 22.0. Student’s *t-*test and one-way analysis of variance were used to compare the groups. A mixed-effects model was applied to assess the impact of multiple factors on axial length change. Fixed effects included baseline characteristics, while random effects accounted for inter-eye differences and duration of Ortho-K lens use. A p-value <0.05 was considered statistically significant.

## RESULTS

Univariate analysis of axial length change in adolescents wearing Ortho-K lenses.

The axial length change was determined as the difference between the initial and final visit measurements. Patients were divided into two groups: those with less than the average axial length change and those with greater than the average change. Differences in age, initial equivalent spherical lens value, initial cylindrical lens value, initial average K value, and initial axial length value were analyzed between the two groups using univariate analysis. The results showed that age (F=-5.476, p<0.001) and initial equivalent spherical lens value (F=8.314, p=0.004) were statistically significant factors influencing axial length change (Table 1).

**Table 1:** Univariate analysis of the influencing factors of wearing an orthokeratology lens on binocular axial length changes in adolescents

Variables		Group with lower-than-average axial length change	Group with higher-than-average axial length change	F- value	p-value
Average corneal curvature	*X ± *S**	43.25 ± 1.24	43.23 ± 1.28	0.017	0.897
	Min-Max	39.85-46.27	40.18-46.37		
	P25-P75	42.43-44.10	42.36-44.13		
	Median	43.47	43.13		
Initial equivalent spherical lens value	*X ± *S**	-3.53 ± 1.18	-2.95 ± 1.42	8.314	0.004
	Min-Max	-6.13-1.00	-5.75-0.50		
	P25-P75	-4.25-2.75	-4.25 -1.78		
	Median	-3.50	-2.50		
Initial axial length value	*X ± *S**	24.96 ± 0.77	24.77 ± 0.82	2.567	0.111
	Min-Max	23.16-27.14	22.87-26.72		
	P25-P75	24.43-25.39	24.12-25.44		
	Median	24.97	24.65		
Initial age	*X ± *S**	12.27 ± 2.17	10.49 ± 1.42	-5.476	<0.001
	Min-Max	8.00-17.00	8.00-15.00		
	P25-P75	11.00-14.00	10.00-11.00		
	Median	12.00	10.00		
Initial cylindrical lens value	*X ± *S**	-0.55 ± 0.50	-0.46 ± 0.40	0.880	0.379
	Min-Max	-2.00-0.00	-1.50-0.00		
	P25-P75	-1.00-0.00	-0.75-0.00		
	Median	-0.50	-0.50		

A p-value of <0.05 was considered statistically significant for comparisons between groups with higheror lower-than-average axial length change.

Univariate analysis of factors influencing axial length changes in adolescents wearing Ortho-K lenses

Univariate analysis was conducted to evaluate the factors influencing axial length changes in the left eye of adolescents wearing orthokeratology (Ortho-K) lenses. The results indicated that age (F=4.132, p<0.001) and initial equivalent spherical value (F=5.840, p=0.018) were statistically significant factors influencing axial length changes between the two groups (Table 2).

**Table 2:** Univariate analysis of the influencing factors of wearing an orthokeratology lens on the axial length change of the left eye in adolescents

Variables		Group with lower-than-average axial length change	Group with higher-than-average axial length change	F-value	p-value
Average corneal curvature	*X ± *S**	43.30 ± 1.28	43.16 ± 1.26	0.261	0.611
	Min-Max	39.85-46.03	40.18-46.37		
	P25-P75	42.20-44.11	42.36-44.07		
	Median	43.61	43.01		
Initial equivalent spherical lens value	*X ± *S**	-3.58 ± 1.28	-2.87 ± 1.41	5.840	0.018
	Min-Max	-6.00-1.00	-5.50-0.50		
	P25-P75	-4.25-2.75	-4.25-1.75		
	Median	-3.63	-2.50		
Initial axial length value	*X ± *S**	24.92 ± 0.79	24.80 ± 0.86	0.455	0.502
	Min-Max	23.16-27.14	22.87-26.52		
	P25-P75	24.40-25.33	24.08-25.56		
	Median	24.97	24.70		
Initial age	*X ± *S**	12.41 ± 2.18	10.48 ± 1.40	4.132	<0.001
	Min-Max	9.00-17.00	8.00-14.00		
	P25-P75	11.00-14.00	10.00-11.00		
	Median	12.00	10.00		
Initial cylindrical lens value	*X ± *S**	-0.67 ± 0.55	-0.58 ± 0.39	-0.663	0.507
	Min-Max	-2.00-0.00	-1.50-0.00		
	P25-P75	-1.00-0.00	-0.75-0.50		
	Median	-0.50	-0.50		

For comparisons between groups with higheror lower-than-average axial length change, a p-value of <0.05 was considered statistically significant.

Univariate analysis of the influencing factors of wearing the Ortho-K lens on the axial length change of the right eye in adolescents

Similarly, univariate analysis was performed to assess factors affecting axial length changes in the right eye of adolescents wearing Ortho-K lenses. The findings revealed that age (F=-3.308, p<0.001) was the only statistically significant factor influencing axial length changes in the right eye (Table 3).

**Table 3:** Univariate analysis of the influencing factors of wearing an orthokeratology lens on the axial length change of the right eye in adolescents

Variables		Group with lower-than-average axial length change	Group with higher-than-average axial length change	F-value	p-value
Average corneal curvature		43.25 ± 1.23	43.27 ± 1.29	0.006	0.938
	Min-Max	40.02-46.27	40.40-46.34		
	P25-P75	42.57-44.12	42.39-44.14		
	Median	43.36	43.17		
Initial equivalent spherical lens value	*X ± *S**	-3.52 ± 1.10	-3.03 ± 1.42	3.227	0.076
	Min-Max	-6.13-1.25	-5.75-0.75		
	P25-P75	-4.25-2.88	-4.25-1.88		
	Median	-3.50	-2.50		
Initial axial length value	*X ± *S**	24.99 ± 0.76	24.75 ± 0.77	2.128	0.148
	Min-Max	23.18-26.82	22.99-26.72		
	P25-P75	24.46-25.49	24.19-25.42		
	Median	24.90	24.65		
Initial age	*X ± *S**	12.13 ± 2.20	10.61 ± 1.53	-3.308	<0.001
	Min-Max	8.00-17.00	8.00-15.00		
	P25-P75	10.00-14.00	10.00-11.25		
	Median	12.00	10.00		
Initial cylindrical lens value	*X ± *S**	-0.43 ± 0.42	-0.35 ± 0.38	0.779	0.436
	Min-Max	-1.50-0.00	-1.25-0.00		
	P25-P75	-0.75-0.00	-0.75-0.00		
	Median	-0.50	-0.25		

For comparisons between groups with higheror lower-than-average axial length change, a p-value of <0.05 was considered statistically significant.

Mixed-effects model analysis of factors influencing axial length changes in adolescents wearing Ortho-K lenses.

To investigate axial length changes from the first to the last visit, a mixed-effects model was established. Fixed effects included sex, initial age, family history, initial equivalent spherical lens value, initial cylindrical lens value, initial average K value, and initial axial length. Random effects comprised the duration of Ortho-K lens wear and inter-eye differences.

The results demonstrated that the difference between the left and right eyes (F=4.94, p=0.0292), the duration of Ortho-K lens wear (F=3.57, p=0.0327), age (F=35.14, p<0.0001), the initial equivalent spherical value (F=4.96, p=0.0278), and the initial axial length (F=205.20, p<0.0001) significantly influenced axial length changes (Table 4). Among these factors, the mixed-effects model indicated that age (t=-5.93, p<0.0001) and initial axial length (t=14.32, p<0.0001) had the most substantial impact (Table 5).

**Table 4:** Test results of the fixed effects in the mixed-effects model

Effects	Type 3 Tests of Fixed Effects					
	Numerator degrees of freedom	Denominator degrees of freedom	Chi-square	F-value	Pr > Chi-square	pr > F
Eye	1	78.2	4.94	4.94	0.0263	0.0292
Group	2	81.9	7.13	3.57	0.0282	0.0327
Sex	1	80.6	0.35	0.35	0.5525	0.5542
Age	1	84.4	35.14	35.14	<0.0001	<0.0001
ESPH	1	119	4.96	4.96	0.0259	0.0278
AK	1	113	0.98	0.98	0.3218	0.3240
FHCATN	1	78.7	0.15	0.15	0.6939	0.6950
BAXISOC	1	104	205.20	205.20	<0.0001	<0.0001

For fixed effects, significance testing was performed by examining the ratio of the estimated value to the standard error (t value). A p-value of <0.05 was considered statistically significant.

**Table 5:** Solutions of the fixed effects in the mixed-effects model

Effects	Solutions of the fixed effects						
	Eye	Number of years of wearing the orthokeratology lens	Estimated value	Standard error	Degree of freedom	t-value	pr > |t|
Eye	0		-2.6431	3.3853	110	-0.78	0.4366
Eye	1		-2.6863	3.3848	110	-0.79	0.4291
Group		1	-0.2707	0.1124	83.2	-2.41	0.0183
Group		2	-0.1841	0.07869	80.8	-2.34	0.0218
Group		3	0				
Sex			-0.03709	0.06245	80.6	-0.59	0.5542
Age			-0.08622	0.01454	84.4	-5.93	<0.0001
ESPH			0.08438	0.03788	119	2.23	0.0278
AK			0.04062	0.04100	113	0.99	0.3240
FHCATN			0.01563	0.03971	78.7	0.39	0.6950
BAXISOC			1.1079	0.07734	104	14.32	<0.0001

AK= initial average corneal curvature; BAXISOC= initial axis length value; ESPH= initial equivalent spherical lens value; FHCATN= family history; group, number of years wearing the orthokeratology lens.

## DISCUSSION

Advances in Ortho-K lens materials have improved oxygen permeability, minimizing hypoxic stress and corneal edema. As a result, overnight lens wear has become a viable method for myopia control^(5^,^6)^. Using this technology, patients can wear specialized lenses overnight to temporarily reshape the cornea and reduce myopia. This treatment allows individuals with myopia to experience significant improvement in their vision upon waking, eliminating the need for corrective lenses throughout the day. Ortho-K lenses offer notable advantages and present an attractive solution for young myopic patients in their daily academic and personal activities^(7^,^8)^. Research has established a strong correlation between the progression of myopia in adolescents and axial length elongation. Slowing the rate of axial length growth is beneficial in mitigating myopia progression^(9)^. Based on these findings, this study aimed to explore the factors influencing axial length changes in adolescents using Ortho-K lenses and to provide a scientific basis for optimizing Ortho-K treatment for myopic adolescents.

In this study, 84 adolescents aged 9-17 yr who were prescribed Ortho-K lenses at our hospital were selected as participants. Data were collected on participants’ gender, age at initiation of Ortho-K lens use, family history of myopia, initial equivalent spherical lens value, initial cylindrical lens value, initial average K value, and initial axial length. A single-factor analysis and a mixed-effects model were employed to assess the factors influencing axial length changes. Based on the average axial length change before and after Ortho-K lens use, participants were classified into two groups: those with below-average axial length changes and those with above-average axial length changes.

The results indicated that for both eyes and the left eye independently, age and initial equivalent spherical lens value were significant factors influencing axial length changes. For the right eye, age was the primary influencing factor. These findings align with previous studies, which suggest that axial length continues to increase with age and that growth accelerates during adolescence, further contributing to axial elongation during this period^(10^,^11)^. To refine the analysis of factors influencing axial length changes following Ortho-K lens use, a mixed-effects model was constructed based on axial length differences between the first and last clinical visits. The results demonstrated that the difference between left and right eyes, duration of Ortho-K lens use, age, initial equivalent spherical lens value, and initial axial length all had a significant impact on axial length changes. The influence of age and interocular differences was consistent with the results of the single-factor analysis. Additionally, the duration of Ortho-K lens use predominantly influenced the treatment cycle. Variability in initial equivalent spherical lens values and axial length values contributed to differences in axial length changes, as the corrective effect of Ortho-K lenses varies with myopia severity.

This study represents the first analysis of factors affecting axial length changes in Chinese myopic adolescents using Ortho-K lenses. Moreover, it innovatively quantifies the influence of specific variables on axial length changes through a mixed-effects model, providing a theoretical foundation for evaluating the efficacy of Ortho-K lenses in clinical practice.

However, this study has several limitations. First, it examined only a subset of potential influencing factors and lacked a normal control group for comparison. Additionally, the limited number of graphical representations made it challenging to comprehensively illustrate the findings. Furthermore, adolescent myopia progression is strongly associated with environmental factors^(12^-^14)^. Future studies should incorporate additional variables such as height, weight, geographic region, near-work activities, screen time, outdoor activity duration, and dietary habits for a more comprehensive analysis. The study also had a relatively small sample size and did not include multivariate analyses. Larger studies with both univariate and multivariate analyses are needed to strengthen the findings.

In conclusion, this study suggests that interocular differences, duration of Ortho-K lens use, age, initial equivalent spherical lens value, and initial axial length value are key factors influencing axial length changes in adolescents wearing Ortho-K lenses. These findings provide valuable theoretical insights for assessing the therapeutic effects of Ortho-K lenses in myopic adolescents, highlighting their potential for broader clinical application.
